# Increased skin blood flow during low intensity vibration in human participants: Analysis of control mechanisms using short-time Fourier transform

**DOI:** 10.1371/journal.pone.0200247

**Published:** 2018-07-12

**Authors:** Yi-Ting Tzen, Eileen M. Weinheimer-Haus, Thomas F. Corbiere, Timothy J. Koh

**Affiliations:** 1 Department of Physical Therapy, University of Illinois at Chicago, Chicago, Illinois, United States of America; 2 Department of Kinesiology and Nutrition, University of Illinois at Chicago, Chicago, Illinois, United States of America; USF Health Morsani College of Medicine, UNITED STATES

## Abstract

**Aim:**

Investigate the immediate effect of low intensity vibration on skin blood flow and its underlying control mechanisms in healthy human participants.

**Materials and methods:**

One-group pre-post design in a university laboratory setting. Nine adults underwent two bouts of 10-minute vibration (30Hz, peak acceleration 0.4g). Outcome measures include skin blood flow, and skin temperature on the right foot. To examine the control mechanisms underlying the vibration-induced blood flow response, SHORT-TIME Fourier analyses were computed to obtain the spectral densities for three frequency bands: metabolic (0.0095–0.02Hz), neurogenic (0.02–0.06Hz), and myogenic (0.06–0.15Hz). Non-parametric Friedman’s tests were computed to compare changes of the outcome measures and control mechanisms over the course of vibration.

**Results:**

Vibration increased skin blood flow during both bouts of vibration, however the effect did not last after vibration was terminated. Myogenic spectral density increased during both bouts of vibration, whereas the metabolic and neurogenic spectral densities increased only during the 2^nd^ bout of vibration. Interestingly, only the metabolic spectral density remained elevated after vibration ended.

**Conclusion:**

Low intensity vibration produced acute increases in skin blood flow mediated in part by vascular control mechanisms of myogenic origin. Further investigation is warranted to determine whether low intensity vibration induces similar increases in skin blood flow in populations prone to developing chronic non-healing wounds, such as spinal cord injury and diabetes.

## Introduction

Chronic wounds, including pressure injuries, diabetic ulcers, and venous ulcers are difficult to treat and can take months or more to heal [1]. Chronic non-healing wounds exhibit chronic inflammation, compromised microcirculatory responses, and low tissue oxygenation [2]. Modality devices are often used to treat chronic wounds as an adjunctive approach to standard wound care, such as ultrasound, negative pressure therapy, diathermy and hyperbaric oxygen therapy [3,4]. These therapies are thought to promote healing by reducing inflammation, enhancing tissue perfusion and oxygenation and inducing a more favorable healing environment.

Recently, low intensity vibration (LIV) has been investigated as a modality to accelerate wound healing [5,6]. A recent study from our group investigated the effect of LIV on wound healing on diabetic mice and demonstrated that LIV increased angiogenesis and granulation tissue formation, and accelerated wound closure and re-epithelialization [5]. In addition, a study on elderly patients with stage I pressure injuries showed that patients that underwent vibration therapy exhibited a greater number of healed ulcers and healing rate compared to those with standard care [6]. Furthermore, a series of studies has shown that skin blood flow (SBF) is increased transiently in the extremities of healthy participants after the application of vibration [7] independent of vibration frequency between 30-50Hz [8]. Thus, it seems reasonable to posit that LIV-enhanced tissue perfusion may promote wound healing.

Despite the positive findings of above published studies of LIV in wound healing and SBF, the underlying beneficial mechanisms have not been fully explored. Two studies suggested that the LIV-enhanced SBF resulted from an increase in endothelial-derived nitric oxide (NO) production [9,10], however other mechanisms independent of endothelial function may also play a role in this phenomenon [9,11]. Previous studies showed that by analyzing skin blood flow signal with time-frequency analyses may reveal corresponding control mechanisms *in vivo* non-invasively [12–14]. These studies were based on the observation that SBF signals acquired with laser Doppler flowmetry system demonstrate oscillatory patterns similar to other cardiovascular signals, such as heart rate variability obtained with electrocardiogram (ECG) [15]. Using heart rate variability as a predicate, previous studies showed that, in addition to time domain parameters of heart rate variability (e.g. interval difference), frequency analysis of R-R intervals provides additional information of autonomic control of the heart [16,17]. Following the same principle of frequency analysis of heart rate variability, researchers also developed time-frequency analyses methods to decompose the rhythmic activities of skin blood flow signal [12,13,18–20]. By analyzing the signal in frequency domain, they found that the skin blood flow signal peaked around the following ranges: 0.005–0.0095 Hz, 0.0095–0.02 Hz, 0.02–0.06 Hz, 0.06–0.15 Hz, 0.15–0.4 Hz, and 0.4–1.6 Hz [12,13,18–20]. These studies found that each frequency interval corresponds with different vascular control mechanism by comparing the SBF signal and ECG and breathing signals in frequency domain, and by observing changes in the peak amplitude with induction or blockage of vascular control pathways [12,13,18–20]. Frequency intervals of 0.4–1.6 Hz and 0.15–0.4 Hz were identified as activities related to heart rate and respiration respectively, since these two peaks were identified in simultaneous measurement of ECG and respiratory activities correspondingly [12]. Frequency range of 0.06–0.15Hz was correlated with blood pressure regulation since this peak was also identified in blood pressure signal. Researchers suggested that this frequency interval corresponds to myogenic control of SBF since smooth muscle cells respond to changes in intravascular pressure [12,13]. Frequency range of 0.02–0.06Hz was identified as neurogenic control of SBF, since this peak attenuated after denervation as well as nerve-blockade [19]. Frequency range of 0.0095–0.02Hz was identified as signals corresponding to endothelial-dependent vascular control activity (metabolic control of SBF), since this peak increased with acetylcholine (Ach) iontophoresis [13]. A very low frequency 0.005–0.0095 Hz was revealed later as SBF control correlated to NO independent endothelial vasodilation (potentially endothelium-derived hyperpolarizing factor), since the peak during this frequency interval increase with Ach iontophoresis but not attenuated with inhibition of NO synthesis [18].

The above described time-frequency analyses of SBF signals have been demonstrated as a promising tool to understand the underlying mechanisms of SBF changes with different stimuli, such as localized heating on healthy [21], and older adults [22], as well as adults with type 2 diabetes [23]. Other studies also investigate localized post ischemic hyperemia on healthy adults and people with spinal cord injury [24–27]. Short-time Fourier transform (STFT) is a form of time-frequency analysis, and we have applied this method previously on SBF data collected in healthy adults and adults with SCI to understand the beneficial effect of local cooling of the skin on ischemic tissue [24,26]. Thus, we adopted the same STFT method to investigate potential mechanisms that may correspond to the changes in SBF with LIV.

The purpose of this study was to investigate the immediate (during the application of LIV) and short-term (after the application of LIV) effects of LIV on SBF, and the underlying vascular control mechanisms using STFT to improve our understanding of the mechanisms by which LIV may help to maintain homeostasis in healthy tissue and help to restore homeostasis following tissue damage. The potential three mechanisms and corresponding frequencies that we computed include: metabolic (0.0095–0.02 Hz), neurogenic (0.02–0.06 Hz), and myogenic (0.06–0.15 Hz). We omitted analysis of frequency intervals correspond to respiration and heart rate since both control mechanisms are unlikely to play a role on local mechanical stimuli such as LIV. We also omitted analysis of frequency interval of 0.005–0.0095 Hz as the NO-independent vasodilation is not as well-defined as the NO-mediated response [15]. This frequency band has not been included as commonly as the other three frequency intervals in previous studies for time-frequency analyses [21–26], and our baseline and post LIV duration is too short to capture changes in this frequency band [18].

## Materials and methods

This study involved two different protocols. The first was used to test the validity of the signal measured by laser Doppler flowmetry (LDF) during LIV, and the second was the main protocol used to test the effects of LIV on SBF and skin temperature.

### Participants

Nine human participants between 30–55 years old were recruited for the study (5 female, 4 male). Written informed consent was obtained for all participants prior to any research procedures. They were all free of peripheral vascular disease, diabetes, and neurological impairment based on self-report medical history. They did not have wounds or any skin disorder at the time of testing as inspected by the authors. Participants reported not taking any medications that might affect SBF (e.g. hypertension medications, beta-blockers), and none of them were smokers at the time of testing. Participants fasted overnight and did not consume any caffeine in the 18 hours prior to the experiments. All procedures involving human participants were approved by the University of Illinois at Chicago institutional review board (# 2013–0536).

### Test setting

**Fig 1:**
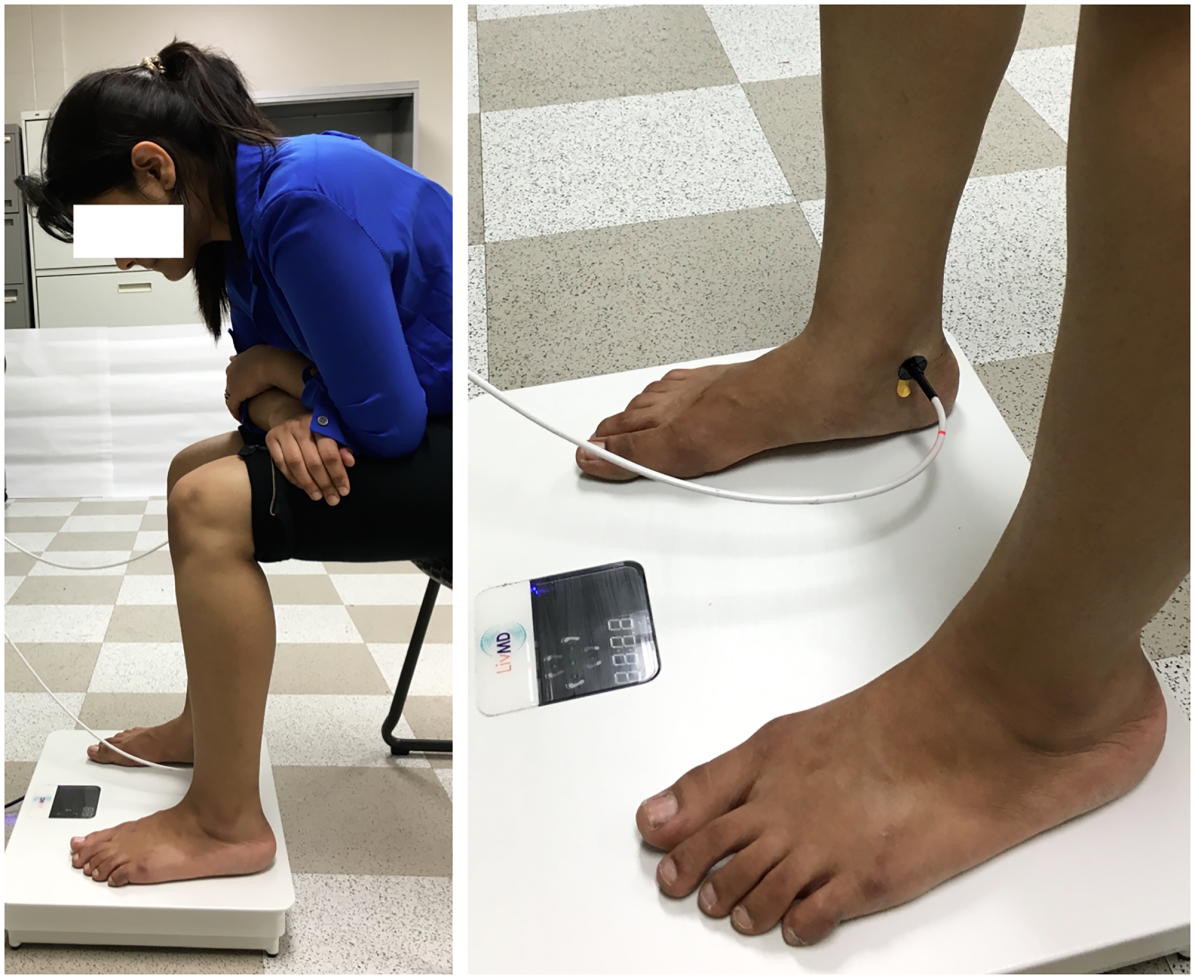
Test setting. (Left) test setting, and (right) probe placement.

The test setting was the same for both protocols. To ensure each participant maintained the same posture throughout the experiment, he/she was asked to sit on a height-adjustable mat table with his/her feet on the LIV platform. The height of the table was adjusted to ensure the angles at ankle and the knee were maintained at 90°. To standardize loading between feet and platform, each participant was instructed to lean on his/her elbows which were placed on the top of the knees. Fig 1 demonstrates the test setting. All participants sat quietly for 10 minutes before the experimental protocol was initiated.

LIV was applied using a platform that vibrated vertically at 30Hz with peak acceleration of 0.4 g (0.22mm peak to peak displacement) for 10 minutes (LiveMD, Marodyne Medical LLC, Tampa, FL). To measure SBF data continuously, an optic probe (CP1T-1000, Moor Instruments, Wilmington, DE) was placed on the right foot. The probe was a combination of laser Doppler flowmetry (LDF) and thermometer. PowerLab 8/35 with LabChart V8 (AD Instruments, Colorado Springs, CO) was used for data acquisition. The probe was placed at the midpoint of the horizontal line drawn between the medial malleolus and the Achilles tendon. Fig 1 demonstrates the placement of the probe for measuring SBF and temperature.

### LDF signal validity test protocol

To test the validity of the LDF signal during LIV, we attempted to block the SBF signal that could be measured by LDF with an opaque surface covering the skin. If the increase in LDF signal with LIV were caused by movement artifact, we would observe similar responses with or without an opaque surface. We had one subject undergo 1 minute of LIV delivered by the platform under two circumstances: with and without blocking the LDF signal with an opaque surface. To create this opaque surface, we placed four layers of white skin tape between the LDF probe and the skin. A piece of paper was inserted between tape layers to ensure blockage of SBF readings. The SBF was collected continuously at 20Hz for one minute before and after the one-minute LIV (total 3 minutes) for both circumstances. The SBF signals were down sampled to 0.5Hz (using MatLab code ‘decimate’), and a spectrogram was computed for the signal (*s*(*τ*)) to obtain spectral densities using Eq 1 [28]. This signal processing method was published previously for analysis of reactive hyperemic acquired on spinal cord injured adults [24].

$$P_{SP}(t,\omega )={\left|S_{t}(\omega )\right|}^{2}={\left|\frac{1}{\sqrt{2\pi }}\int e^{-i\omega \tau }s(\tau )h(\tau -t)d\tau \right|}^{2}$$ (1)

Spectrogram is the magnitude-square of STFT, *S*_*t*_(*ω*). A 128-second Hanning window (*h*(*τ*)) was selected to ensure good time resolution of SBF signal over the course of LIV of the validity protocol. MatLab code ‘spectrogram’ was used to compute spectrogram. To confirm that the signal recorded during LIV was valid, we compared the spectral densities at the following three time points: 0.5^th^, 1.5^th^, and 2.5^th^ minute with and without opaque surface (representing the time intervals of baseline, LIV and post LIV). If the spectral densities were similar at the 1.5^th^ minute (during LIV) with and without the opaque surface blocking the signal, this would indicate artifactual signal.

### LIV main experiment protocol

**Fig 2:**
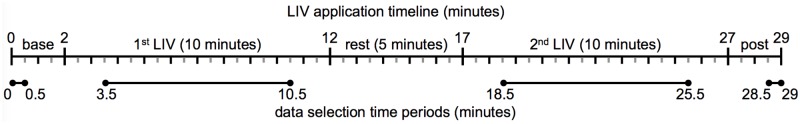
Study timeline. Timeline of LIV application and SBF data collection, and timeline for data selection for statistical analyses.

For the main experiment protocol, we utilized a one-group pretest-posttest design. All nine participants underwent the same two bouts of vibration protocol, and outcomes were collected for comparison before vibration (baseline), during 1^st^ and 2^nd^ vibration and post vibration (post). To test the effects of LIV on SBF and skin temperature, the main experimental protocol involved: LIV delivered by the platform for 10 minutes (first bout), then 5 minutes of rest, followed by a second bout of LIV for 10 minutes. The second bout of LIV was implemented to determine whether the effect of LIV on SBF was more pronounced with a second bout. SBF was collected continuously at 20Hz for two minutes before, and after the experimental protocol (total 29 minutes, see illustration in Fig 2). To determine whether skin temperature was affected by LIV, or vice versa, skin temperature was also measured. Because skin temperature did not fluctuate as much as the SBF data, only one data point was taken at the end of each of the four time intervals: two minutes of baseline, 10 minutes of 1^st^ LIV, 10 minutes of 2^nd^ LIV, and two minutes post LIV.

### Data analyses

To analyze the SBF signals collected from the LIV main experimental protocol, the SBF signals were down sampled to 0.5Hz using MatLab code ‘decimate’. Spectrogram was calculated using STFT (Eq 1) to obtain spectral densities of the down-sampled SBF signal. A 512-second Hanning window (*h*(*τ*)) was selected to ensure that the spectral main lobe of the window was smaller than the narrowest physiological band of our interest (0.095–0.02Hz) [24]. To compare the changes in spectral densities within each frequency band across the course of vibration, we summed up the spectral densities within each of the three frequency bands and the four time intervals for statistical analyses. The three frequency bands and its corresponding vascular control mechanisms are: metabolic (0.0095–0.02Hz), neurogenic (0.02–0.06Hz), and myogenic (0.06–0.15Hz) mechanisms; the four time intervals and its corresponding periods are: baseline (0–0.5^th^ minute), 1^st^ LIV (3.5–10.5^th^, minute), 2^nd^ LIV (18.5–25.5^th^ minute), and post LIV (28.5-29^th^ minute). This four time periods were selected to ensure that we do not include spectral densities during the transition phase between each interval for comparison. Eq 2 demonstrated the calculation of spectral densities for statistical analyses, where ω represents the frequency range, and *T* represents the time interval of interests. In addition, to ensure the spectral densities among time intervals are comparable, integral of spectral densities within each time period was divided by the amount of SBF signal sampled within that time period (n). For example, to calculate the metabolic spectral density during baseline, the following parameters were used in Eq 2: ω1 = 0.0095Hz, ω2 = 0.02Hz, *T*1 = 0 second, *T*2 = 30 seconds, and n = 15; to calculate the myogenic spectral density during 2^nd^ LIV, the following parameters were used: ω1 = 0.06Hz, ω2 = 0.15Hz, *T*1 = 1110 seconds, *T*2 = 1530 seconds, n = 210.

$$P_{-T\omega }=\left(\int_{\omega =\omega 1}^{\omega 2}\int_{T=T1}^{T2}\left(T,\omega \right)dTd\omega \right)/n$$ (2)

To ensure our comparison of SBF for each time interval is consistent with the selection for spectral densities, the SBF was averaged during the above mentioned time periods for statistical analyses. Fig 2 illustrates the selection of the four time periods. We used MatLab program (The Mathworks, Natick, MA, USA) to down sample the signal, and compute STFT. The above mentioned signal processing method was published previously for analyzing SBF signal collected on adults with spinal cord injury [24].

Due to the small sample size of this pilot study (single group, n = 9), nonparametric tests for repeated measures were computed for statistical analyses. Friedman tests were used to compare the changes of SBF, temperature, and spectral densities with LIV application. *Post hoc* analyses were computed by taking the absolute value of the difference between the mean ranks of comparisons [29]. This absolute value of difference for each comparison was then compared to the critical difference as shown in Eq 3. The critical difference for our comparison is 1.61, where *Z*_*α*/*k*(*k*-1)_ is 2.64 (calculated with α level of 0.05, and k is 4 time intervals), k is 4, and N is 9 subjects. Difference between mean ranks is reported for all *post hoc* analyses.

$$|{\overset{-}{R}}_{u}{-\overset{-}{R}}_{v}|\geq z_{\alpha /k(k-1)}\sqrt{\frac{k(k+1)}{6N}}$$ (3)

Due to the small sample size, the effect size (*r*) for each pair of comparison was calculated in addition to *p* value. Effect size is the ‘magnitude of the difference between groups’ [30], which is the magnitude of the difference in SBF between comparisons (over the course of LIV) in our case. Calculating and reporting effect size could help us understand the changes in SBF over the course of LIV despite the small sample size. Effect size is computed from the *z*-score of the Wilcoxon signed-rank test using formula $z/\sqrt{\left(N\right)}$, where N is the number of observation, which is 18 in our calculation (9 observations during each time interval). Absolute value of effect size <0.3 is considered small, whereas >0.5 is considered large. SPSS ver22 (IBM Corp., Armonk, NY) was used for all statistical analyses.

## Results

### SBF signal is not significantly influenced by motion artifact during vibration

**Fig 3:**
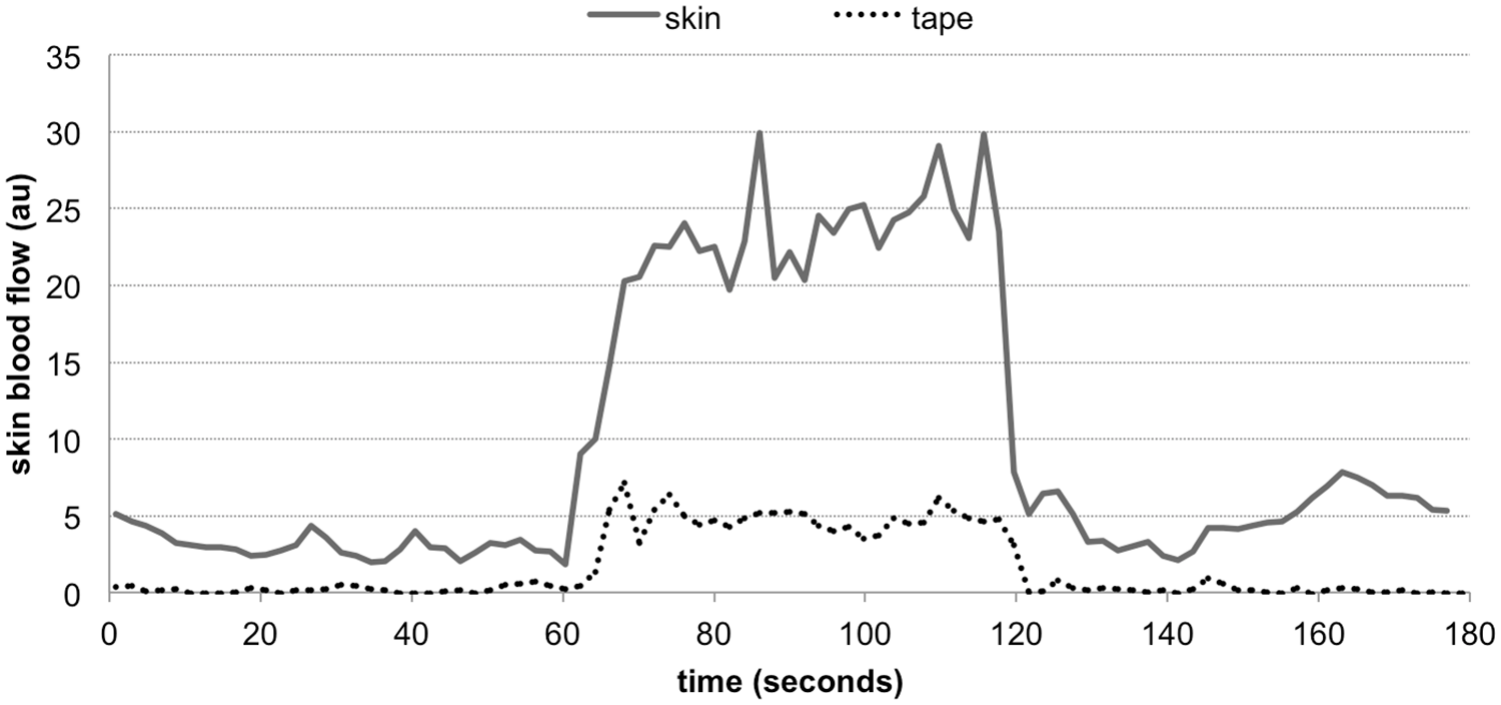
Skin blood flow artifact check. Skin blood flow signals collected with and without tape blocking the signal. LIV was applied during 60–120 seconds.

**Fig 4:**
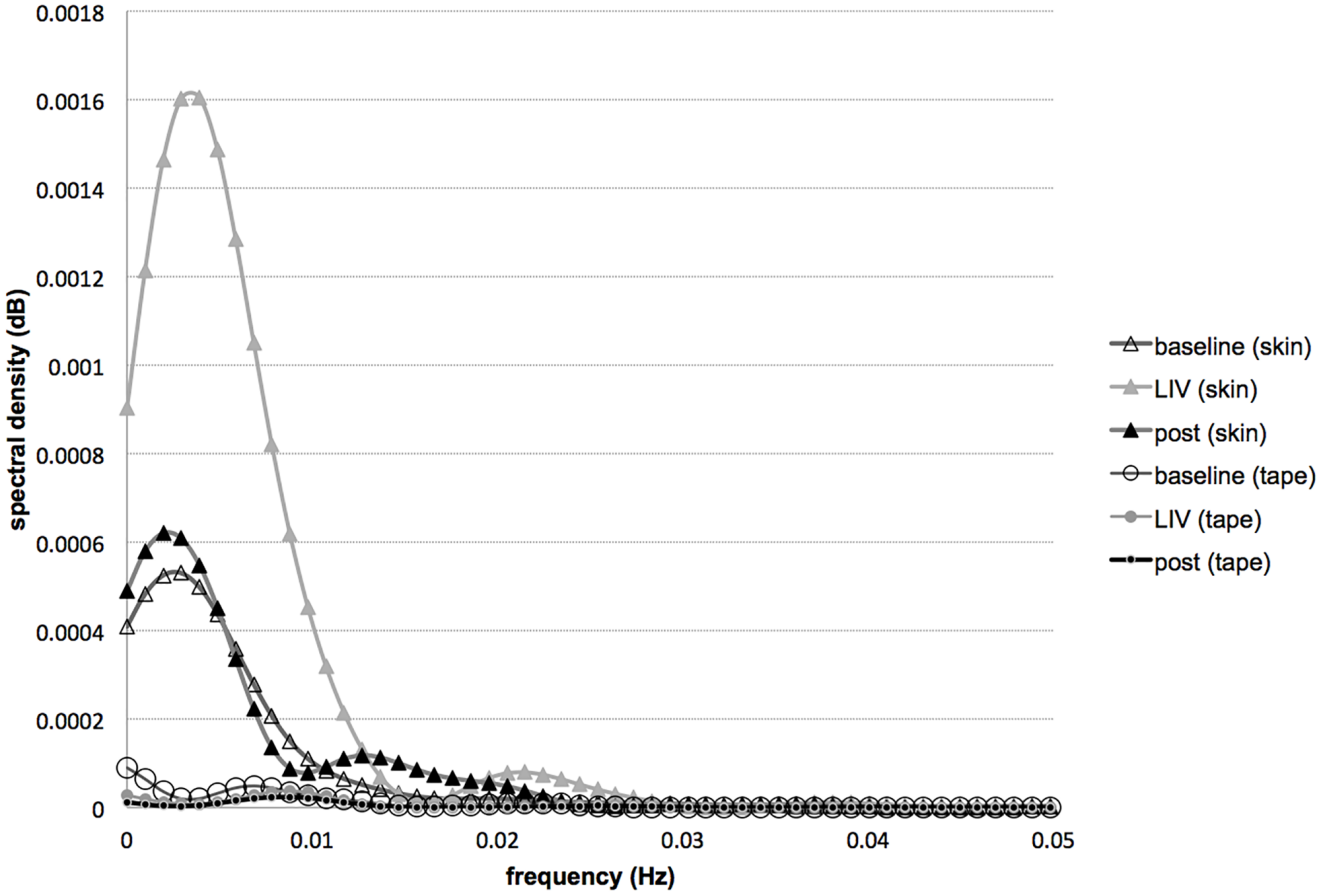
Skin blood flow artifact check with STFT. Spectral densities of LDF signal on skin and tape during baseline (before LIV application), LIV, and post LIV.

Fig 3 shows SBF data collected with and without the skin covered with an opaque surface. There was only a minor increase in signal (<5 au) with LIV when the skin is covered, while the increase in signal with LIV when the skin was not covered was approximately 20–40 au. Short-time Fourier analyses showed that the difference in spectral densities among baseline, vibration and post vibration was very minor when the skin was covered (Fig 4). These methods suggested that the increase in SBF with LIV measured in this study is unlikely affected by movement artifact induced by probe vibration.

### LIV induces immediate increase in SBF

**Fig 5:**
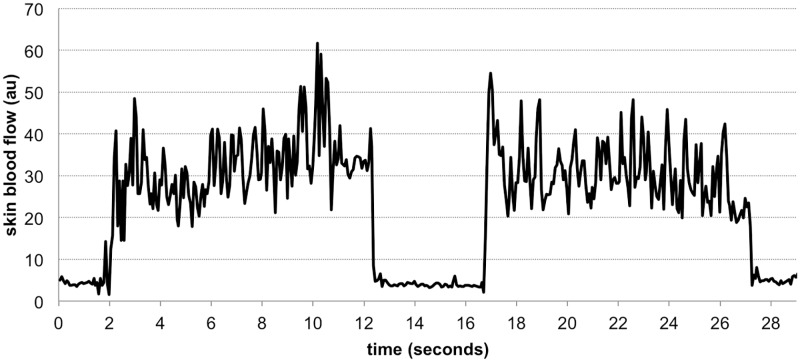
Example of down-sampled SBF signals at foot. This example is collected from subject #2.

**Fig 6:**
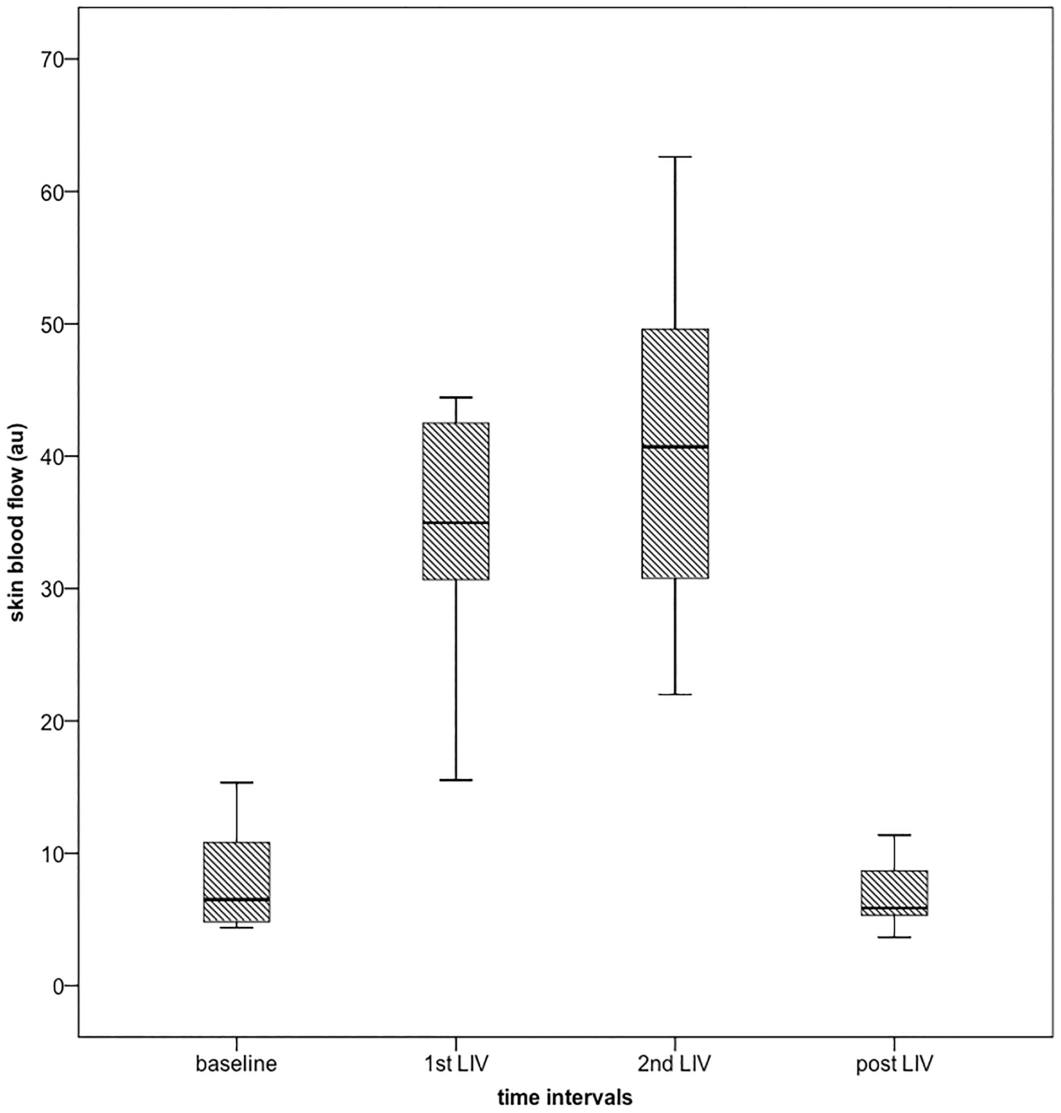
Boxplots of mean SBF during the four time intervals at foot (n = 9). Subject # 9 is an outlier (beyond 1.5 interquartile range) during 1^st^ and 2^nd^ bout of LIV (250 and 350 au, respectively, not shown on graph).

**Fig 7:**
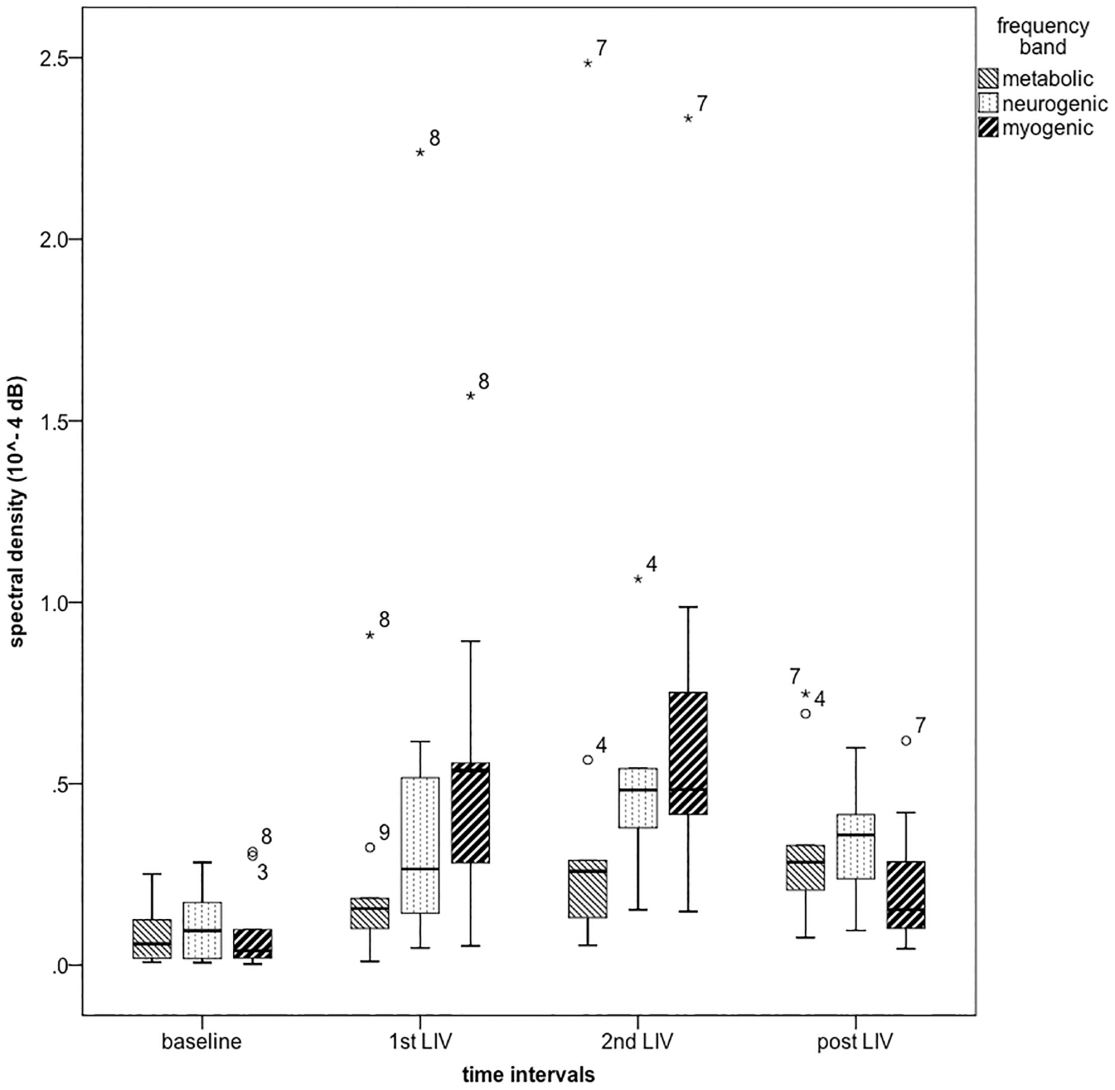
Boxplots of spectral densities at foot (n = 9). Outliers (beyond 1.5 interquartile range) are marked with subject ID.

We observed an immediate, dramatic, increase in SBF with both bouts of vibration in all nine subjects. Fig 5 demonstrates a typical SBF signal at the ankle, and Fig 6 shows boxplots of averaged SBF before (baseline), during both bouts of vibration (1^st^ and 2^nd^ LIV), and post vibration (post LIV). Fig 7 shows boxplots of averaged spectral densities during the above-mentioned four time intervals. Table 1 includes the mean ± one standard deviation of SBF, spectral densities and skin temperature during baseline, 1^st^ LIV, 2^nd^ LIV, and post LIV. Table 1 also includes mean ranks of each comparison as computed by Friedman tests, and the statistical significance as compared to baseline value computed with *post hoc* analyses are also indicated at α level of 0.0083 (using Bonferroni correction of 0.05/6 pairs). Table 2 shows the effect size of each pair-wise comparison.

**Table 1:** SBF, spectral densities and skin temperature at foot.

Outcome measures (unit) (mean rank)	Time Intervals			
	Baseline	1^st^ LIV	2^nd^ LIV	Post LIV
Skin blood flow (au)	8.34 ± 4.05 (1.56)	59.05 ± 77.92 ^a^ (3.44)	77.88 ± 117.69 ^a^ (3.56)	6.90 ± 2.55 (1.44)
Metabolic spectral density (10^−6^ dB)	8.41 ± 8.04 (1.78)	22.59 ± 27.13 (2.56)	47.23 ± 77.07 (2.67)	33.26 ± 23.47 (3.00)
Neurogenic spectral density (10^−6^ dB)	11.13 ± 10.38 (1.56)	50.16 ± 68.03 (2.67)	79.65 ±100.86 ^a^ (3.33)	32.30 ± 16.33 (2.44)
Myogenic spectral density (10^−6^ dB)	9.58 ± 12.29 (1.11)	53.61 ± 46.93 ^a^ (3.33)	70.01 ± 66.57 ^a^ (3.33)	22.19 ± 18.89 (2.22)
Skin temperature (°C)	26.34 ± 1.62 (3.78)	25.98 ± 1.54 (2.89)	25.51 ± 1.70 ^a^ (1.89)	25.39 ± 1.73 ^a^ (1.44)

Mean ± one standard deviation of SBF, spectral densities and skin temperature at foot during the four time intervals. Mean ranks of each comparison are included in parentheses.

^a^ The difference as compared to baseline value reaches statistical significance (*p*<0.0083)

**Table 2:** Effect size (*r*) of each pair-wise comparison.

Outcome measures	Comparison			
	Baseline—1^st^ LIV	1^st^ LIV—2^nd^ LIV	Baseline—2^nd^ LIV	Baseline—Post LIV
Skin blood flow	-0.63	-0.29	-0.63	-0.24
Metabolic spectral density	-0.45	-0.10	-0.65	-0.77
Neurogenic spectral density	-0.46	-0.15	-0.60	-0.49
Myogenic spectral density	-0.63	0.07	-0.63	-0.46
Skin temperature	-0.60	-0.01	-0.63	-0.60

Effect size of each comparison in SBF, spectral densities and skin temperature over time intervals. Absolute value of effect size >0.5 is considered large.

Friedman test showed that there was significant difference in SBF over the four time intervals (χ^2^(3) = 21.667, *p*<0.001). *Post hoc* analyses showed that SBF was increased during both bouts of LIV as compared to baseline, however this effect did not last after LIV was terminated. There was no difference between the two bouts of LIV. The myogenic spectral density behaved similarly to the SBF. Friedman tests showed that there was significant difference in myogenic (χ^2^(3) = 18.333, *p*<0.001) spectral densities among the four time intervals. *Post hoc* analyses showed that the myogenic spectral density was increased during both bouts of LIV as compared to baseline. The increase in myogenic spectral density did not last during post LIV, and there was no difference between the two bouts of LIV. Calculation of effect size as demonstrated in Table 2 confirmed the above findings suggesting that the increase in SBF and myogenic spectral density is large during both bouts of vibration but not after LIV ended.

We also found that the neurogenic spectral density was significantly different among the four time intervals (χ^2^(3) = 8.733, *p* = 0.033), and was significantly greater during the 2^nd^ LIV as compared to baseline. Effect size calculation suggested that the difference in neurogenic spectral density was large between the baseline and 2^nd^ LIV. Similar to the myogenic spectral density, the increase in neurogenic spectral density did not last after LIV ended. Friedman test showed that there was no difference in metabolic spectral density across four time intervals (χ^2^(3) = 4.333, *p* = 0.228). However, the effect size calculation suggested that the difference in metabolic spectral density between baseline and 2^nd^ LIV (*r* = -0.65), and between baseline and post LIV (*r* = -0.77) was large. Neither of these two comparisons (baseline-2^nd^ LIV: *p* = 0.051, baseline-post LIV: *p* = 0.021) reached statistical significance (*p* < 0.0083) due to small sample size of this pilot experiment [30].

For the skin temperature, there was a significantly difference among the four time intervals (χ^2^(3) = 18.929, *p*<0.001). The skin temperature during the 2^nd^ LIV, and post LIV were significantly lower than that during baseline. Effect size calculation suggested that the differences in skin temperature between baseline and 1^st^ LIV, baseline and 2^nd^ LIV, and baseline and post LIV are large; however, the gradual decrease in skin temperature over time is between 0–1.7°C (mean ± one standard deviation: 0.96 ±0.58 °C).

## Discussion

The primary findings of this study are that LIV applied to the bottom of the foot induces an immediate increase in SBF at the ankle, remains elevated throughout LIV application and returns to baseline after LIV is terminated. For this study, we performed continuous measurement of SBF during the course of LIV application, whereas previous studies that measured SBF before and after application of vibration. A previous study suggested that vibration may induce artifact in LDF signals measured during application of vibration [31] and to our knowledge all studies published thus far have measured SBF immediately before and after vibration [7,9,11,32] or during temporary cessation of vibration over the course of vibration [8]. We believe our measurements reflect true changes in SBF since we showed that LIV applied to the measurement probes (with blocked SBF signal) did not produce significant LDF signal in and of itself. In addition, the vibration amplitude used in our protocol (peak acceleration 0.4 g, 0.22 mm peak to peak displacement) was much smaller than those used in previous studies (peak acceleration 7g, 5-6mm [7–9,32], and 2mm [11]). Thus, our findings suggest that use of LIV allows measurement of SBF in real time with LDF, which showed immediate increase in SBF with application of LIV.

To investigate the potential vascular control mechanisms that underly increased SBF with LIV, we computed STFT of SBF signals. Our findings suggested that the 1^st^ bout of LIV-induced changes in SBF at the foot is mainly mediated by myogenic regulation, whereas the 2^nd^ bout of LIV-induced changes in SBF at foot is partially mediated by metabolic, neurogenic and myogenic regulation. In addition, the metabolic regulation of SBF seemed to remain increased after LIV ended as suggested by the large effect size demonstrated in Table 2. Previous studies suggested that the metabolic regulation of SBF is likely the NO-dependent endothelial vasodilatory response [12,21], since this peak in time-frequency analysis demonstrated an increase with Ach iontophoroesis [13]. Our results showed that this response appeared to increase during the 2^nd^ bout of LIV and persisted after LIV ended. Our data complements findings from previous studies which reported that the increase in SBF post vibration may be caused by shear stress elicited NO production [9,10]. This shear stress induced NO production helps increase blood flow, and may be a local effect [11]. Our findings suggested that, with the use of STFT, we were able to identify when this response occurred even there was no significant changes in SBF after LIV ended.

Importantly, our findings suggested that NO production is not likely the only factor contributing to vibration increased SBF. Our results suggested that other factors likely also contribute to the increased SBF response, including neurogenic and myogenic regulation. Previous studies showed that the neurogenic control mechanism is likely ganglionic nerve controlled vasomotion [19], which is the vasodilatory response caused by release of acetylcholine and secretory neuropeptides [33]. Our finding may also help explain why the previous study found that diabetic patients demonstrate a similar rate of NO production but lower blood flow response with vibration as compared to healthy adults [9]. In addition, another mechanism independent of NO production has been suspected to contribute to the increase in blood flow response with vibration in people with type 2 diabetes [11]. Since changes in autonomic neuropathy have been reported secondary to diabetes [33], it is possible that a diabetes-induced impairment in the neurogenic vasodilatory response may cause a smaller LIV-induced SBF response. Our STFT analyses suggested that neurogenic response took effect during 2^nd^ bout of LIV but did not last after LIV ended. Future studies on LIV and STFT analyses of SBF signal in subjects with diabetic neuropathy or spinal cord injury with autonomic impairments may help to clarify the mechanisms of LIV-induced SBF changes.

Previous studies suggested that the frequency interval of 0.06–0.15 Hz was related to myogenic regulation of SBF to maintain intravascular pressure [12]. It is suggested that this myogenic regulation is vasoconstriction induced by changes in blood vessel transmural pressure, and it may be a local mechanism that is stress dependent to counterbalance the sudden increase in SBF [34]. Our STFT analyses suggested that myogenic regulation contributed during both bouts of LIV, and we speculate that this vasoconstriction was induced to maintain transmural pressure during the mechanical stress of vibration. The underlying endothelium dependent vasodilation warrants further investigation in studies involving patient with endothelial dysfunction, such as diabetes. Investigation of very low frequency 0.005–0.0095 Hz with longer time of SBF collection may be beneficial in understanding the NO-independent endothelial vasodilation.

Another main finding of this study is that the increase in the SBF did not last after vibration was terminated. This finding is not consistent with previous studies, which demonstrated an elevated SBF response up to 10 minutes after termination of vibration [7,32]. We suspected that reason for the lack of lasting effect with our LIV protocol is the lower amplitude of our vibration protocol than that of previous studies. The vibration parameters used in previous studies were 30-50Hz, 5–6 mm amplitude, 6–7 g peak acceleration, where as our LIV parameters were 30 Hz, 0.2 mm amplitude, 0.4 g peak acceleration.

To determine whether a change in skin temperature may have contributed to the SBF response, skin temperature was measured. We did not find any increase of skin temperature over the course of the study, indicating that an increase in skin temperature was not responsible for the increased SBF induced by LIV. This is consistent with a previous study, which showed that, although vibration has a positive effect of increasing SBF, vibration reduces the risk of burn as compared to methods utilizing thermal energy [32]. In fact, our results demonstrated a small gradual decrease in skin temperature at the ankle over the course of vibration application, likely due to the exposure of the skin to the air which temperature (21°C) was lower than body temperature. Since this decrease in skin temperature is very small (<1.7°C), it likely did not affect the SBF signal. We suggest that the skin and probe should be covered with light clothing in the future studies with similar protocol to prevent this small decrease in skin temperature.

A limitation of this study was that our non-invasive measurements could only examine superficial tissue (up to 2–3 mm deep), therefore findings of our study cannot be generalized to deeper tissues, including muscles. Another potential limitation of this study was that in the seated position adopted by the participants of this study, gravity may have influenced both the transmission of LIV signals and the physiological responses to them. In addition, since this same position was used for all subjects, the primary outcome is unlikely to be affected by the posture. We understand from previous studies that anthropometric measurements (such as body mass index, skin fold thickness), gender and menstrual cycle may play a role in SBF regulation, however due to the exploratory nature of this study we did not collect this information [35,36]. Nevertheless, since we compare the changes in SBF within each subject, we believe such factors do not confound our primary finding that LIV acutely increases blood flow. A study investigating whether obesity or diabetes influences the blood flow response to LIV is planned for the future. Several previous studies examined the influence of whole body vibration on leg blood flow using Doppler ultrasound either acutely [37–41] or after 12 weeks of training [42]. These previous studies also demonstrated increased femoral artery blood flow with acute bouts of vibration or after weeks of training. Since the vibration parameters, measurement tool, and the target blood vessel (artery vs. capillary) were different, comparisons between these studies and our current study are difficult. Lastly, STFT results do not provide direct physiological measurements (e.g. changes in vessel diameter, production of NO, or NO-independent vasodilatory substances), however this method could serve as a non-invasive proxy for these measurements *in vivo*.

## Conclusion

Findings from our study demonstrate that LIV causes an immediate increase in SBF that returns to baseline after LIV is terminated. STFT analyses of SBF suggested that a myogenic mechanism contributes to both bouts of LIV, whereas metabolic and neurogenic mechanisms contribute to 2^nd^ bout of LIV. The metabolic mechanisms persist after LIV ends, at which time SBF does not remain elevated. In the future, we plan to compare LIV-induced SBF responses between non-diabetic and diabetic subjects and LIV-induced responses in patients with chronic wounds, including pressure injuries and diabetic ulcers and will probe the mechanisms by which LIV induces SBF at the tissue, cell and molecular level.
